# The Efficacy of Microfracture Combined with Extracorporeal Shock Wave Therapy for Treating Osteochondral Lesion of the Talus and the Quality of Regenerated Cartilage: A Retrospective Cohort Study and MRI Assessment

**DOI:** 10.3390/jcm12082966

**Published:** 2023-04-19

**Authors:** Jian Li, Qiaozhi Ma, Jianlei Hou, Yufen Liu, Pengfei Lu, Pengwei Liu, Zhongwen Zhang, Gengyan Xing

**Affiliations:** 1 Department of Orthopaedics, China Aerospace Science & Industry Corporation Hospital 731, Beijing 100074, China; 13501091665@139.com; 2 Department of Orthopedics, The Third Medical Center of PLA General Hospital, Beijing 100039, China; 3 Department of Radiology, The Third Medical Center of PLA General Hospital, Beijing 100039, China; 4 Department of Orthopedics, Changshou People’s Hospital of Chongqing, Chongqing 401220, China

**Keywords:** osteochondral lesion of the talus, extracorporeal shock wave therapy, T2 mapping, cartilage regeneration, hyaline-like cartilage, ankle function

## Abstract

Background: osteochondral lesion of the talus (OLT) is a common disease in the physically active population, and extracorporeal shock wave therapy (ESWT) is a noninvasive treatment. We hypothesized that microfracture (MF) combined with ESWT may have great potential to become a novel combination treatment of OLT. Methods: the OLT patients who received MF + ESWT or MF + platelet-rich plasma (PRP) injection were retrospectively included, with a minimal follow up of 2y. The daily activating VAS, exercising VAS, and American Orthopedic Foot and Ankle Society Ankle-Hindfoot Score (AOFAS) were used to assess the efficacy and functional outcome, and ankle MRI T2 mapping was used to evaluate the quality of regenerated cartilage in the OLT patients. Results: only transient synovium-stimulated complications were found during the treatment sessions; the complication rate and daily activating VAS did not have differences between groups. MF + ESWT had a higher AOFAS and a lower T2 mapping value than MF + PRP at the 2y follow up. Conclusions: the MF + ESWT had superior efficacy for treating OLT, which resulted in better ankle function and more hyaline-like regenerated cartilage, superior to the traditional MF + PRP.

## 1. Introduction

Osteochondral lesion of the talus (OLT) refers to talar cartilage detachment with or without a subchondral bone fragment [1], which is a common disease in the physically active population. The chondral layer of the talus is quite vulnerable to injury since (one) the self-healing ability of cartilage is naturally limited [2], and (two) the talus dome has no soft tissue (ligament or tendon) attachment, making the blood supply of this area relatively limited. Hence, the cartilage has very limited self-healing ability after injury. The pathological process of OLT involves both the articular cartilage and the underlying subchondral bone.

OLT patients usually need operative interventions. According to Hepple’s classification [3], OLT patients with stage III~IV lesions and those who failed the conservative treatment are suggested to receive operative interventions [4]. Microfracture (MF) is a subchondral bone marrow stimulation technique, which has been generally considered the most common first-line operative intervention for OLT [5]. However, it has been well known that MF leads to cartilage repair with fibrocartilage, mechanically inferior to hyaline cartilage [6]. Hence, MF has been clinically used as the basic intervention in a combination treatment for OLT, for example, the widely used combination of MF and platelet-rich plasma (PRP) injection, and evidence suggested that it is effective for treating OLT [5,7]. Extracorporeal shock wave therapy (ESWT) is a newly applied noninvasive treatment for OLT, which has been proven to be beneficial in osteoarthritis (OA), such as knee OA (KOA) [8,9], hip osteonecrosis [10], and OLT [4]. ESWT has the following advantages: noninvasive, does not require hospitalization, and is low cost. An in vivo study found that ESWT can induce neovascularization and upregulate angiogenesis and osteogenesis-related growth factors in the osteochondral lesion model [11]. Hence, we hypothesized that MF combined with ESWT may have great potential to become a novel effective treatment of OLT.

Present clinical studies on MF combined with ESWT for treating cartilage lesions mainly focus on KOA. The study on OLT is very rare and limited, lacking longitudinal clinical studies and systematic comparisons. The indication, safety, efficacy, and functional outcomes of MF combined with ESWT in treating OLT remain unknown. The present study aims to compare the safety, efficacy, functional outcomes, and regenerated cartilage quality between MF + PRP and MF + ESWT and investigate the affecting factor for the outcomes of cartilage repair in OLT patients. The minimum follow up was two years (y).

## 2. Methods

### 2.1. Subjects

This is a retrospective cohort study. The OLT patients who received the combination treatment of MF and PRP or ESWT from March 2017 to August 2020 in our department were continuously included in this study. Inclusion criteria: (1) symptomatic OLT, age: 18–45 years old, BMI ≤ 31; (2) OLT ≤ 15 mm^2^ [12,13,14,15], International Cartilage Repair Society (ICRS) grade 3~4 by the arthroscopic examination (grade 3: 100% ≥ defect > 50% of the cartilage thickness; grade 4, a full-thickness OLT revolving the subchondral bone); (3) unilateral OLT; (4) preoperative and postoperative MRI were presented; and (5) minimum follow up >24 months. Exclusion criteria: (1) subchondral bone cyst; (2) MRI showed at least 2 ligament ruptures of the ankle joint, or notable ankle instability; (3) accompanied with fracture nonunion, muscular dystrophy, vascular injury, and nerve injury of the ankle joint; (4) lower extremity malalignment [16]; and (5) diabetes, rheumatoid arthritis, ankylosing spondylitis, and Parkinson’s disease. 

The OLT patients were divided into the PRP group and ESWT group according to their received treatment. The OLT patients were well informed about both the combination treatment simultaneously, including the advantages (e.g., MF + PRP can produce a reliable short-term efficacy; ESWT is a noninvasive treatment) and disadvantages (e.g., the long-term efficacy of MF + PRP may be controversial; the efficacy of MF + ESWT for treating OLT remains unclear), and the patients made the final decision based on their wills. The retrospective observational study was conducted in accordance with the Declaration of Helsinki for protecting human subjects and approved by the Ethics Committee of Third Medical Center of PLA General Hospital (IRB 2019-03-01-ob). 

### 2.2. General Characteristics 

Basic characteristics included: age (years), sex, BMI, disease history, ICRS grade of OLT, the surface area of OLT, follow up time, and complications during the treatment sessions. The complications consisted of fever, pain, hematoma, edema, arthritis, and synovitis of the ankle joint, as well as operative infection, deep vein thrombosis, nerve injury, and limited range of motion (ROM). All of the data above was extracted from the medical record system.

Enrolled in this study were 76 patients with a minimal follow up of 2y. The MF + PRP group (n = 33) consisted of 14 military soldiers, 9 professional athletes, 5 college students, 2 manual workers, and 3 sports teachers/coaches. The MF + ESWT group (n = 43) consisted of 22 military soldiers, 11 professional athletes, 6 college students, 2 manual workers, and 2 sports teachers/coaches. Most of the OLT patients had a definite exercising injury history associated with training or exercise (58/76), such as ankle sprain and edema. The general characteristics did not have a difference between the 2 groups (Table 1). 

During the PRP treatment sessions (MF + PRP group), 1 patient had a transient low fever (37.5 °C~37.9 °C) and symptomatic ankle synovitis (synovitis= pain + edema), and 3 patients had transient ankle pain (Table 1); during the ESWT treatment sessions (MF + ESWT group), 2 patients had transient symptomatic ankle synovitis, 3 patients had transient ankle pain, and 7 patients had transient asymptomatic edema (Table 1). In the present study, “transient” ankle pain and “transient” edema mean that these symptoms temporarily occur in the acute phase after the treatment, and last less than 3 days with or without NSAIDs. All of the patients who had the complications above were allowed to use 500 mg of acetaminophen up to 3 times/day for less than 3 days if their symptoms were unbearable for the patients. The complication rate of the MF + PRP group had a lower trend compared with the MF + ESWT group (Table 1). Although more edema cases were found in the MF + ESWT group than in the MF + PRP group (7 cases vs. 0), the total complication rate had no statistical difference between the 2 groups (Table 1).

### 2.3. Arthroscopic MF Operation

All of the operations were performed by one senior surgeon. The surgical procedure was carried out after spinal anesthesia with the patient in a prone position. The thigh tourniquet was inflated at 280 mmHg to 300 mmHg pressure on the affected side. 

The ankle was kept in a neutral position, and ankle traction was performed with a sterilized foot strap. Anteromedial and anterolateral portals of ankle arthroscopy were established by the standard technique. First, an arthroscopic examination was performed using the anteromedial and anterolateral portals alternately, and the location of osteochondral lesions was determined. To obtain an adequate visualization, ankle tractions in dorsal extension or plantar flexion were essential. Second, arthroscopic debridement and lesion curettage were performed to remove the necrotic tissues until the underlying hard subchondral bone (viable) was exposed. Third, an MF was performed by using 40° and 90° MF awls, knocking into the subchondral bone plate at the depth of 3 to 4 mm, and the MF operations were performed 3 to 4 mm apart from each other [10,17]. Releasing the tourniquet, marrow fat droplets and blood flowing out of the MF holes were observed. The operation was finished, and the lower extremity was immobilized with a high polymer cast (short cast) in a neutral position.

### 2.4. PRP Injection

PRP injection was started on the next day of MF operation [18]. Each patient in the PRP group received 3 injections separated by 3 months intervals. 

PRP was prepared using a GPS III Platelet Separation System (Biomet Biologics, Warsaw, IN, USA), and the protocol of producing PRP was performed according to the system instructions. A total of 70 mL of venous blood was drawn from each patient in this group, mixed with 5 mL of citrate for the inhibition of clotting. The total solution of 75 mL was centrifuged for 15 min at 4 °C, 3200 r/min. At the end of the procedure, 4 mL of PRP was obtained. A 22-gauge needle was used, and the posterolateral approach was used for the joint injection [19]. We recommended the patients do an ankle flexion–extension motion to cover the PRP solution evenly. Daily rehabilitation included full ROM movement and isometric and isotonic contraction of the tibialis anterior muscle and posterior muscle, however, passive stretch and full weight bearing were prohibited on the first day after PRP injection.

### 2.5. ESWT

**Figure 1:**
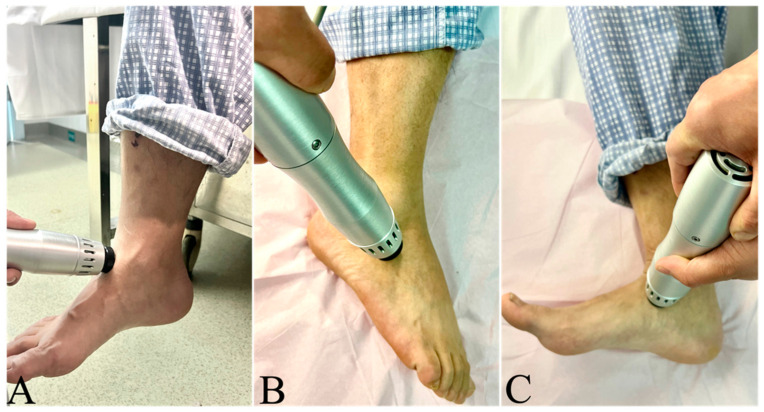
OLT patients’ position during ESWT (**A**) ESWT was applied in a neutral position when the OLT was in the talus dome; (**B**) the patient was in a lateral decubitus position with ankle flexion when the OLT was in the lateral surface of the talus; (**C**) the patient was in a lateral decubitus position with ankle extension when the OLT was in the medial surface of the talus.

ESWT augmentation to MF was started 3 months postoperation of MF, and all of the ESWTs were performed by an experienced therapist using the Electromagnetic Shock Wave Emitter (EMS DolorClast, Electro Medical Systems, Switzerland). The lower extremity was placed in an appropriate position according to the OLT area (Figure 1), and the ultrasound gel was applied as a contact medium between the device and the patient’s skin to minimize the energy loss of the shock wave. The ESWT selected 2 to 3 treatment points around the osteochondral defects according to the MRI findings.

The parameters of ESWT were set as follows [10,20]: (1) energy flux density, 0.14 to 0.16 mJ/mm^2^ (3~3.5 bar, 8 Hz); (2) frequency, 40~50 times/min; and (3) the number of impulses, 2000 impulses/treatment. 

Each session contained 5 times of ESWT separated by one day, and each OLT patient received 3 treatment sessions at intervals of 2 months. The schedule of ESWT was listed as the following. Session 1: 1st month (Day 1: ESWT; Day 3: ESWT; Day 5: ESWT; Day 7: ESWT; Day 9: ESWT), Session 2: 3rd month (Day 1: ESWT; Day 3: ESWT; Day 5: ESWT; Day 7: ESWT; Day 9: ESWT), and Session 3: 5th month (Day 1: ESWT; Day 3: ESWT; Day 5: ESWT; Day 7: ESWT; Day 9: ESWT). The rehabilitation protocol was carried out during the ESWT session, the same as the one during the PRP treatment session.

### 2.6. Subjective Assessments 

All patients were assessed with the visual analogue scale (VAS) and American Orthopedic Foot and Ankle Society Ankle-Hindfoot Score (AOFAS) before and at the follow up. The pain of OLT was assessed by VAS during both daily activities and exercise. The AOFAS was used to assess the pain, activity capacity, range of motion, gait, and alignment of the ankle, giving general information about the ankle’s overall function [21]. The total of AOFAS is 100 scores, and a higher score represented a better ankle function.

### 2.7. Successful Rate of the Treatments

Successful treatment was assessed by meeting 3 of the following criteria at the follow up [15]: (1) more than 50% improvement in VAS for pain during daily activities; (2) more than 50% improvement in VAS for pain during exercise; (3) AOFAS was increased by at least 30 points or reached 100 points at the follow up.

### 2.8. MRI T2 Mapping for the Outcomes of Cartilage Repair

MRI T2 (transverse relaxation time) mapping is a novel technique for the compositional assessment of articular cartilage repair and regeneration [9,22]. An increased T2 mapping value is thought to be a marker of cartilage degeneration [6,9], while a decreased T2 mapping value in the follow up is thought to be a marker of regenerated hyaline-like cartilage in the outcome [9]. 

Quantitative cube T2-mapping examinations of OLT patients were performed by a senior physician using a 3T MRI unit (GE DISCOVERY MR750) with a surface coil wrapped around the ankle joint. The patients were imaged in a supine position, with the ankles positioned neutrally and the legs straight. T2-mapping was conducted using the following parameters: (1) the repetition time was set to 1000 ms, and the echo time was set to 32 ms; (2) the slice thickness was set at 3.5 mm with a spacing of 0.7 mm; (3) the field of view was set to 256 mm × 256 mm; (4) the number of excitations was 1. The T2 mapping was performed before treatment and at the follow-up point of a minimum of 2y postoperation.

### 2.9. Statistical Analysis

The continuous data were expressed as mean ± SD, and the count data were expressed as number (n) and rate (/). The comparisons of continuous data were processed by the independent *t*-tests and Levene variance homogeneity tests, while the comparisons of the count data were processed by the Chi-square test or Fisher’s exact test. Multiple linear-regression analysis was performed to determine the independent factors of the efficacy in each combination treatment for OLT. The level of significance was set at 0.05. All the statistical analyses were performed using IBM SPSS 20.0.0 (SPSS Inc., 2009, Chicago, IL, USA). 

## 3. Results

### 3.1. Comparisons of the Efficacy

Before the combination treatments, the daily activities VAS, exercise VAS, and AOFAS of the OLT patients did not have a significant difference between the MF + PRP and MF + ESWT group at baseline (Table 2).

At the follow up, the daily activities VAS and the successful rate did not have a significant difference between the MF + PRP and MF + ESWT groups at the follow up (Table 2). The exercise VAS of the MF + PRP group was lower than that of the MF + ESWT group, while the AOFAS of the MF + ESWT group was significantly higher than that of the MF + PRP group (Table 2).

### 3.2. MRI T2 Mapping for the Cartilage Repair

**Figure 2:**
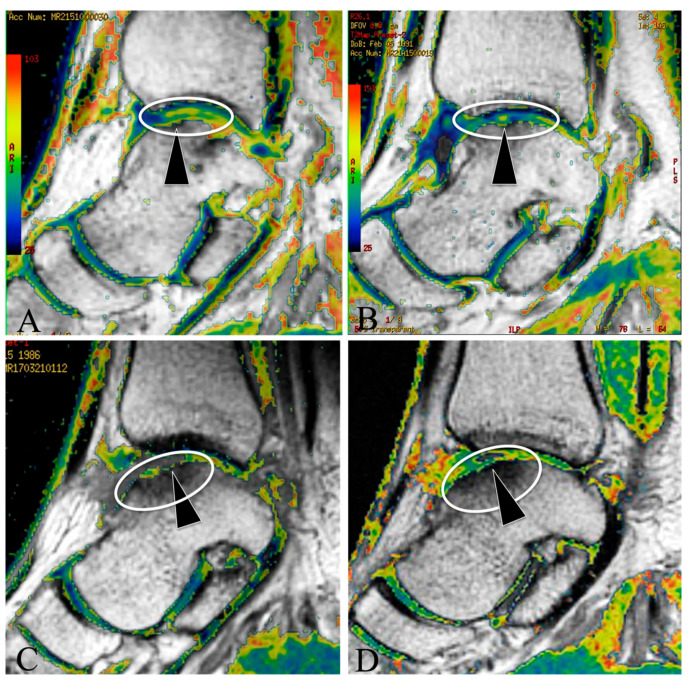
MRI T2 mapping assessment on the cartilage quality in MF + ESWT and MF + PRP groups at baseline and 2y follow up (**A**) baseline T2 mapping for OLT patients in the MF + ESWT group, the ellipse showed an OLT area (yellow-green, high T2 mapping value), and the arrow showed an osteochondral lesion in a patient; (**B**) T2 mapping for the MF + ESWT group at 2y follow up, the ellipse showed the regenerated cartilage (blue-green, lower T2 mapping value) in the same OLT patient at 2y follow up, and the arrow showed the osteochondral lesion had been refilled with neo-cartilage tissue; (**C**) baseline T2 mapping for OLT patients in the MF + PRP group, the ellipse showed an OLT area (yellow, high T2 mapping value), and the arrow showed a cartilage defect in a patient; (**D**) T2 mapping for the MF + PRP group at 2y follow up, the ellipse showed immature regenerated cartilage (green-yellow, slightly lower T2 mapping value) in the same OLT patient at 2y follow up, and the arrow showed the osteochondral lesion had been refilled with immature neocartilage tissue.

Baseline MRI T2 mapping for the patients showed evidence of OLT in both groups (Figure 2A,C), and the T2 mapping values of OLT areas did not have a significant difference between the MF + PRP and MF + ESWT groups at baseline (Table 3). 

At the 2y follow up, MRI T2 mapping showed a neo-regenerated cartilage tissue in all of the patients of the two groups (Figure 2B,D), however, it showed that some of the osteochondral lesion in the MF + PRP had been refilled with immature neocartilage tissue (with a green color) (Figure 2D), while the osteochondral lesion in the MF + MF + ESWT had been refilled with a relatively mature neocartilage tissue (with a blue-green color) (Figure 2B), and the T2 mapping value of the MF + ESWT group was lower than that of the MF + PRP group (Table 3). 

### 3.3. Multiple Linear-Regression Analysis 

We selected the potential affecting factors (age, sex, BMI, disease history, ICRS grade, and OLT area) and the different treatment methods (one for MF + PRP, two for MF + ESWT) to determine the independent factors of the regenerated cartilage quality in OLT patients. The multiple linear-regression analysis showed that the treatment method (B = −6.951) and age (B = 0.393) were the independent factors affecting the outcome of regenerated cartilage in OLT patients (Table 4). 

## 4. Discussion

OLT is susceptible to sports injury and it can be caused by a single or repetitive trauma, such as an ankle sprain and fracture. In the present study, most of the OLT patients had a definite exercise-injury history associated with training or exercise (58/76). It has been reported that OLT can be found in 70% of ankle sprains and fractures [23,24]. OLT is very common among athletes, affecting about 5.2 per 10,000 athletes [23,24]. In the present study, the average age of OLT patients was 33.9y and 34.4y in the two groups, and the number of male patients was 3.2 times that of the females, suggesting that the physically active population such as young and middle-aged male subjects are relatively susceptible to OLT.

Synovial symptoms were the main complications of MF + PRP and MF + ESWT for treating OLT. The present study found that the complications in the two groups were similar, including synovitis (pain + edema), simple pain, and simple edema of the ankle joint, and disappeared without any sequelae after orally taking NSAIDs. As OLT patients are always concomitant with chronic synovitis, the joint injection or ESWT may stimulate the synovium tissue, irritating the ankle synovitis. The present result also found that the complication rate of the MF + ESWT had an increasing trend towards the MF + PRP group, for example, the edema (seven cases vs. zero), though no statistical difference was observed between the two groups. ESWT is a pulsed sound wave characterized by a short duration, high-pressure amplitude, and relatively low tensile wave [25]. It is a mechanical energy occurring at the interface of different-density tissues. ESWT may stimulate the synovium tissue, and it has been reported that ESWT can cause transient soft-tissue swelling or minor bruising for treating KOA [9], which may explain the edema after ESWT. However, we did not face any severe complications during the treatment sessions of the two groups. In summary, the present study suggested that both the MF + PRP and MF + ESWT had superior safety for treating OLT, only transient synovium-stimulated complications were found during the treatment sessions.

The present study systematically compared the efficacy between the MF + PRP and MF + ESWT for treating OLT patients. Our results found that the MF + PRP group had a lower exercising VAS compared with the MF + ESWT group, indicating that MF + PRP had a benefit in pain relief. A systematic review has concluded that PRP had a superior efficacy in alleviating pain in OLT patients as an adjunct to MF [5]. We considered that this pain-relieving benefit was associated with the anti-inflammatory effects of PRP. PRP has been suggested to directly modulate pain and reduce synovial inflammation in a recent study [26]. IL-1β and TNF-α are well-known inflammatory factors associated with the joint pain of OA, it has been reported that PRP can inhibit IL-1β and TNF-α to counteract the cartilage catabolic process in many degenerative cartilage diseases [27,28]. An in vivo study also found that PRP-injected knees can decrease the severity of synovitis (thinner synovial membrane) with more anti-inflammatory cells (CD206þ and CD163þ) in a KOA model [29]. The direct effects of reducing ankle synovitis inflammation and the associated pain may explain the pain-relieving benefit of MF + PRP for treating OLT patients in the present study. However, we did not find a significant difference in the daily-activating VAS between the two groups at the 2y follow up. It has been reported that OLT-caused ankle pain may not be notable during rest and daily activities, rather than upon weight bearing and exercising [30], which may explain the reason above. 

The present study found that the MF + ESWT group had a higher AOFAS compared with the MF + PRP group at the minimal 2y follow up, indicating that MF + ESWT can obtain a better overall function of the ankle than MF + PRP for treating OLT. OLT has a series of symptoms including ankle pain, reduced range of motion, impaired function, stiffness, functional instability, swelling, and locking [30], which can be classified into two categories consisting of pain and joint dysfunction. The AOFAS was used to assess the ankle’s overall function of OLT, not only the pain but also the joint functions including the activity capacity, range of motion, gait, and alignment [21]. Although the MF + PRP had a benefit in pain relief for treating OLT, the ankle overall function of MF + ESWT was higher finally. Our results reveal that MF + ESWT can result in better ankle functions than MF + PRP, which is probably beyond the advantage in pain relief of MF + PRP. MF + ESWT consequently results in a higher AOFAS (better overall function) than MF + PRP for treating OLT. We considered that the superior efficacy of ankle function on OLT can be attributed to the ESWT of the combination. We consider that there are two potential mechanisms explaining the benefit of MF + ESWT in AOFAS improvement for treating OLT: (one) ESWT produces a reflexive analgesic effect by inducing axon excitability and destroying the unmyelinated sensory fibers in the target tissue [31]; and (two) ESWT promotes microcirculation, inducing neovascularization (blood supply) in the osteochondral lesion. 

Blood supply is an essential factor directly associated with cartilage regeneration. The present used MRI T2 mapping to evaluate the regenerated cartilage quality in OLT patients, and our results found that the T2 mapping value of the MF + ESWT group was significantly lower than that of the MF + PRP group at the follow up. T2 mapping is a useful predictor of cartilage degeneration and regeneration quality [8,10]. In the early stage of cartilage repair, the neocollagen structure and tissue anisotropy led to an increase of the free water content in the cartilage [8], which is positively correlated with the T2 mapping value on the MRI [32,33], while in mature cartilage (hyaline cartilage), the water protons are immobilized by the collagen–proteoglycan matrix, resulting in an attenuation of the T2 mapping value (low signal intensity) [10]. Hence, T2 mapping can reflect the cartilage regeneration quality after treatments [32], and the lower the T2 mapping value, the closer the regeneration gets to the hyaline-like cartilage. Our results suggested that the cartilage regeneration of MF + ESWT resulted in better quality and was much more mature than MF + PRP, which was much more similar to hyaline-like cartilage. We consider that ESWT-associated neovascularization is responsible for the regenerated hyaline-like cartilage in OLT patients. It has been found that ESWT can induce neovascularization, and up-regulate angiogenesis-related growth factors in an osteochondral lesion model [11], such as the vascular endothelial growth factor [34]. Clinical trials also support the opinion that ESWT-associated neovascularization plays a critical role in the cartilage repair process, for example, avascular osteonecrosis of the proximal femur [10] and KOA [9], and ESWT can rapidly normalize the MRI appearance in these patients [9]. Similar to our results, a pilot study also found that MF + ESWT can significantly improve AOFAS and reduce OLT areas on the MRI at 1y follow up [4]. Hence, it is reasonable to speculate that ESWT-associated neovascularization contributes to cartilage regeneration in OLT patients. What is more, our multiple linear-regression analysis also showed that the treatment method (B = −6.951) and age (B = 0.393) were the independent factors affecting the T2 mapping values of the OLT patients, suggesting MF + ESWT and young age were the independent factors associated with the regeneration of hyaline-like cartilage in the outcome. The present study suggested that MF + ESWT resulted in more hyaline-like cartilage in OLT patients, superior to traditional MF + PRP.

Our study had several limitations. First, as a retrospective clinical study, the selection bias of grouping was inevitable. The OLT patients were well informed about both of the two treatments and made the final decision based on their wills, and the patients’ subjective choices may affect the present results. Second, we did not perform the second look and biopsy to assess the regenerated cartilage quality in the OLT patients. Further RCTs and prospective cohort, as well as second look and biopsy, are needed to determine the macro- and micromorphology changes of the regenerated cartilage in OLT patients treated with MF + ESWT.

## 5. Conclusions

Microfracture combined with extracorporeal shock wave therapy had good safety for treating OLT, only transient synovium-stimulated complications were found during the treatment sessions. Microfracture, combined with extracorporeal shock wave therapy, had superior efficacy for treating OLT, the minimal 2y follow up found that it resulted in better ankle function and more hyaline-like regenerated cartilage that was superior to traditional microfracture combined with PRP injection, however, the two treatments had comparable efficacy on pain caused by daily activity in those patients.

## Figures and Tables

**Table 1:** Basic characteristics of the OLT patients in the MF + PRP and MF + ESWT groups.

Characteristics	MF + PRP (n = 33)	MF + ESWT (n = 43)	*p* Value
Age (year)	33.9 ± 6.8	34.4 ± 7.5	*t* = −0.337 *p* = 0.737
Sex (n)	male 26 female 7	male 32 female 11	*χ*^2^ = 0.197 *p* =0.657
BMI	25.99 ± 2.74	24.86 ± 2.89	*t* = 1.742 *p* = 0.086
Disease history (month)	4.2 ± 3.2	5.0 ± 5.0	*t* = −0.778 *p* = 0.439
ICRS grade (n)	grade 3 15 grade 4 18	grade 3 24 grade 4 19	*χ*^2^ = 0.802 *p* = 0.370
OLT area (mm^2^)	135.61 ± 5.62	136.54 ± 5.51	*t* = −0.721 *p* = 0.473
Follow up (month)	28.9 ± 5.7	27.4 ± 3.0	*t* = 1.447 *p* = 0.155
Complications (n)	no 29 synovitis 1 ankle pain 3	no 31 synovitis 2 ankle pain 3 edema 7	*χ*^2^ = 2.799 *p* = 0.094

Note: MF, microfracture; PRP, platelet-rich plasma; ESWT, extracorporeal shock wave therapy; ICRS, International Cartilage Repair Society; OLT, osteochondral lesion of the talus.

**Table 2:** Comparisons of the efficacy in the MF + PRP and MF + ESWT groups.

Efficacy Parameter	MF + PRP (n = 33)	MF + ESWT (n = 43)	*p* Value
Baseline daily activity VAS	4.1 ± 0.2	4.0 ± 0.6	*t* = 0.790 *p* = 0.433
Daily activity VAS	1.2 ± 0.4	1.3 ± 0.4	*t* = −0.439 *p* = 0.662
Baseline exercise VAS	6.4 ± 0.8	6.5 ± 0.6	*t* = −0.391 *p* = 0.697
Exercise VAS	2.4 ± 0.6	2.8 ± 0.4	*t* = −2.634 *p* = 0.011 *
Baseline AOFAS	65.8 ± 8.0	63.7 ± 14.8	*t* = 0.731 *p* = 0.467
AOFAS	94.0 ± 5.3	96.7 ± 4.2	*t* = −2.371 *p* = 0.021 *
Successful rate (n)	Success 14 Unsuccess 19	Success 26 Unsuccess 17	*χ*^2^ = 2.438 *p* = 0.184

Note: MF, microfracture; PRP, platelet-rich plasma; ESWT, extracorporeal shock wave therapy; VAS, visual analogue scale; AOFAS, American Orthopedic Foot and Ankle Society Ankle-Hindfoot Score; *, *p* < 0.05.

**Table 3:** Comparisons of the T2 mapping values in the MF + PRP and MF + ESWT groups.

T2 Mapping Value	MF + PRP (n = 33)	MF + ESWT (n = 43)	*p* Value
Baseline	39.73 ± 3.26	39.81 ± 3.67	*t* = −0.107 *p* = 0.915
Follow up	23.29 ± 4.43	16.53 ± 2.90	*t* = 8.025 *p* < 0.001 **

Note: MF, microfracture; PRP, platelet-rich plasma; ESWT, extracorporeal shock wave therapy; VAS, visual analogue scale; AOFAS, American Orthopedic Foot and Ankle Society Ankle-Hindfoot Score; **, *p* < 0.01.

**Table 4:** Multiple linear-regression analysis for the T2 mapping value of regenerated cartilage of OLT.

Parameters	B	SEM	*p*-Value	VIF
Treatment	−6.951	0.547	0.000 **	1.052
Age	0.393	0.037	0.000 **	1.530
Sex	0.424	0.756	0.577	1.155
BMI	0.114	0.098	0.252	1.142
Disease history	0.060	0.065	0.359	1.132
ICRS grade	0.391	0.553	0.481	1.538
OLT area	0.085	0.059	0.152	1.052

Note: R^2^ = 0.810; SEM = standard error of mean; VIF = variance inflation factor; **, *p* < 0.01.

## Data Availability

All of the data have been showed in this paper. There are no additional unpublished data from this study. Requests to access these datasets should be directed to the first author (Jian Li, 13501091665@139.com).
